# Primary intraventricular synovial sarcoma of the brain with recurrence - case presentation

**DOI:** 10.1186/s12883-022-02975-w

**Published:** 2022-12-01

**Authors:** Anna McCool, Clinton Turner, Sarah Turner, Peter Heppner, Frank Saran

**Affiliations:** 1 Department of Radiation Oncology Cancer & Blood Research, Auckland City Hospital, Level 12, Building 1, 1023 Auckland, New Zealand; 2 Anatomical Pathology, Auckland City Hospital, Labplus, Auckland, New Zealand; 3 Department of Neurosurgery, Auckland City Hospital, Auckland, New Zealand

**Keywords:** Case report, Neuro-oncology, Synovial sarcoma

## Abstract

**Background:**

We report a case of recurrent primary intraventricular synovial sarcoma of the brain with no extracranial primary, initially reported as a haemangiopericytoma. We believe this is the first reported case of primary intraventricular synovial sarcoma at this site.

**Case presentation:**

A 27-year-old male presented to hospital with a new onset of seizures. Imaging revealed a left ventricular trigone mass with surrounding oedema. He underwent a left occipito-temporal craniotomy and resection with the histology reported as haemangiopericytoma. Resection was followed by adjuvant radiation treatment. Seven years later follow-up imaging revealed a 4 mm contrast enhancing lesion in the previous surgical bed. The patient underwent resection. Histological analysis of the recurrence revealed a spindle cell tumour with a *SS18* gene rearrangement consistent with synovial sarcoma. Retrospective fluorescent in-situ hybridisation analysis of original histology also revealed a *SS18* gene rearrangement consistent with a diagnosis of synovial sarcoma.

**Conclusion:**

Synovial sarcoma should be included as part of the differential diagnosis for patients presenting with intraventricular spindle cell tumours in the brain.

## Background

Synovial sarcoma (SS) is a soft tissue sarcoma that mainly occurs as a deep soft tissue tumor of the extremities. The incidence peaks in the fourth decade with a median age at diagnosis of 35, and has a slight male predominance [1]. It frequently presents as a localized disease in the extremities, especially near large joints such as the knee [2]. Intracranial disease, which is rare, has been reported as a metastasis or as a primary dural tumour [3]. We report a case of intraventricular synovial sarcoma with no obvious primary extracranial pathology, suggesting a primary intraventricular tumour. We describe potential diagnostic pitfalls with synovial sarcoma in the central nervous system (CNS) and the management of recurrence with surgery and adjuvant radiation retreatment.

## Case report

**Fig. 1:**
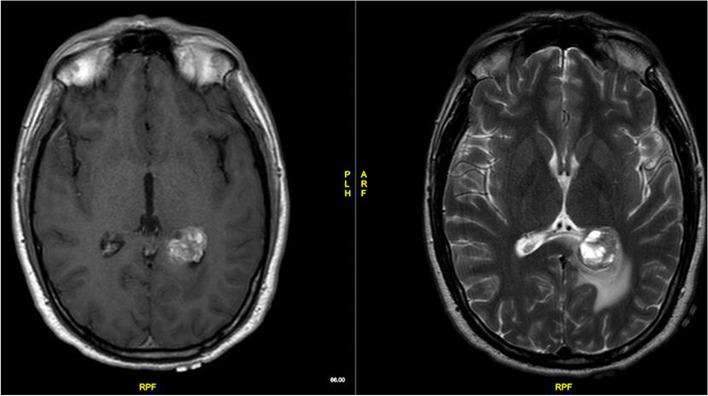
Pre-operative T1 post gadolinium contrast and T2 axial MRI in 2013. There is a 22 mm x 32 mm contrast enhancing circumscribed mass at the atrium of the left lateral ventricle surrounded by oedematous adjacent cerebral parenchyma. The lesion shows mixed solid and cystic components. It appears related to the local choroid plexus. Mild linear enhancement of the ependymal surface of the left occipital horn is suggestive for local infiltration outside of the lesion

A 27-year-old male presented to hospital in 2013 with a new onset of seizures. Magnetic Resonance Imaging (MRI) demonstrated a 3 cm mass within the left ventricular trigone with surrounding oedema (Fig. 1). He underwent a left occipito-temporal craniotomy and resection with the histology reported at this time as haemangiopericytoma, World Health Organization (WHO) grade 2. He received post-operative adjuvant radiation therapy (RT) 54 Gy in 30 fractions using a 3D conformal technique. At the time, given the histological diagnosis, no further staging scans were requested.

**Fig. 2:**
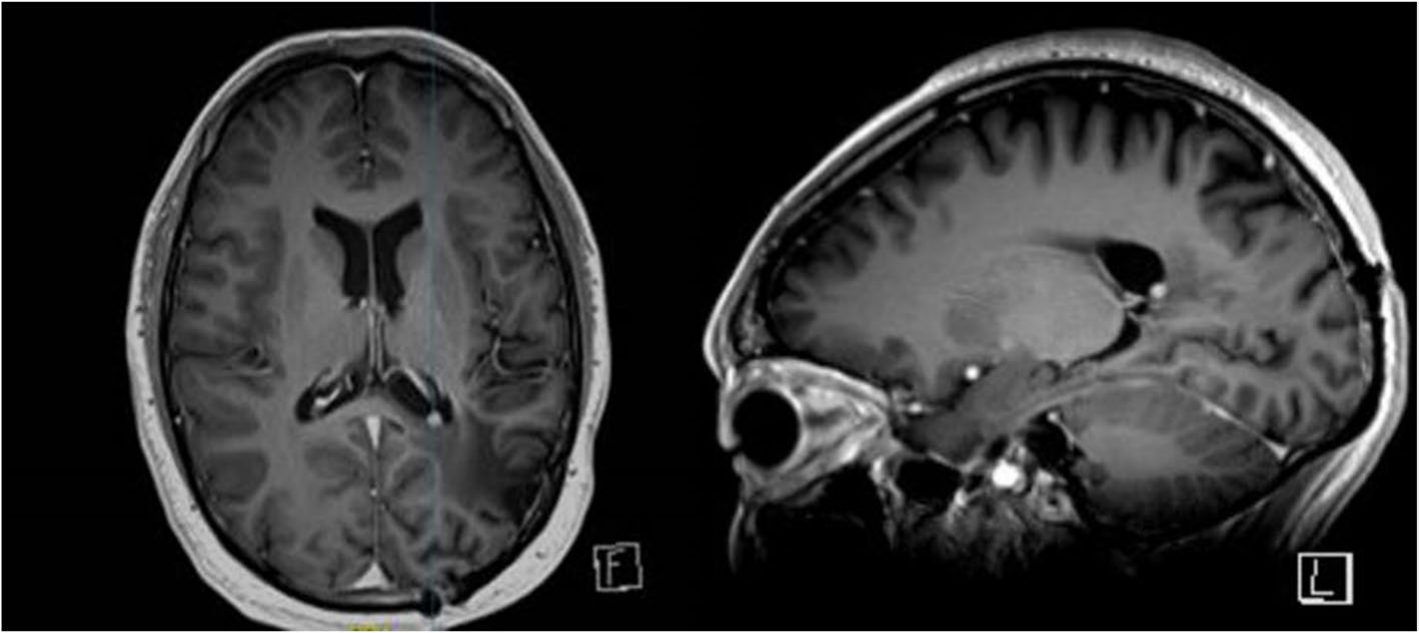
Surveillance MRI 7 years later showing a 4 mm homogenous contrast enhancing lesion within the left ventricular trigone, without restricted diffusion

**Fig. 3:**
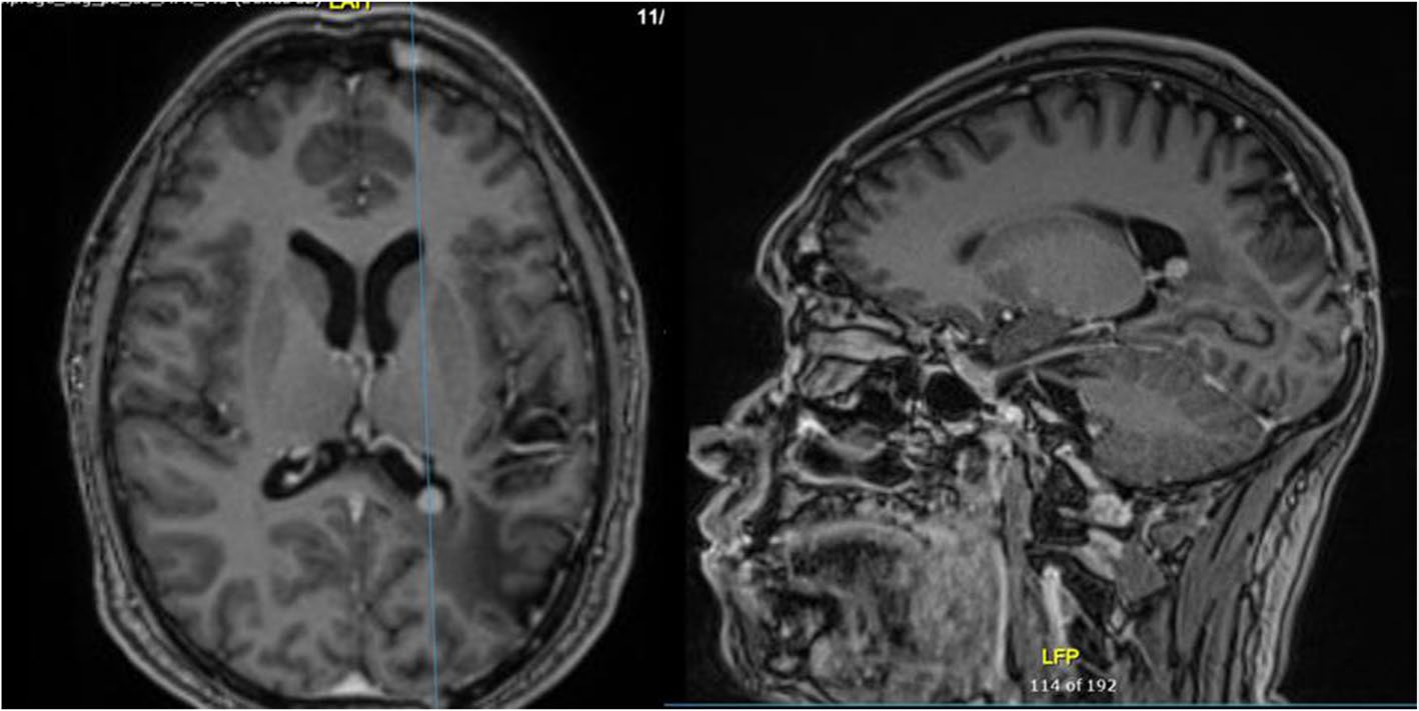
3 months later further increase in size of left ventricular trigone lesion to 8 mm with the same signal characteristics as previously

Follow up included an MRI brain every 6 months for two years then yearly MRI brain scans. Surveillance MRI 7 years after completion of treatment showed a 4 mm contrast enhancing lesion within the left ventricular trigone in the previous surgical bed (Fig. 2). The patient was asymptomatic at the time. The radiological features were thought to be consistent with recurrence and an early follow up interval scan was requested. MRI brain and spine was repeated 3 months later and demonstrated an increase in size of the intraventricular lesion to 8 mm (Fig. 3).

**Fig. 4:**
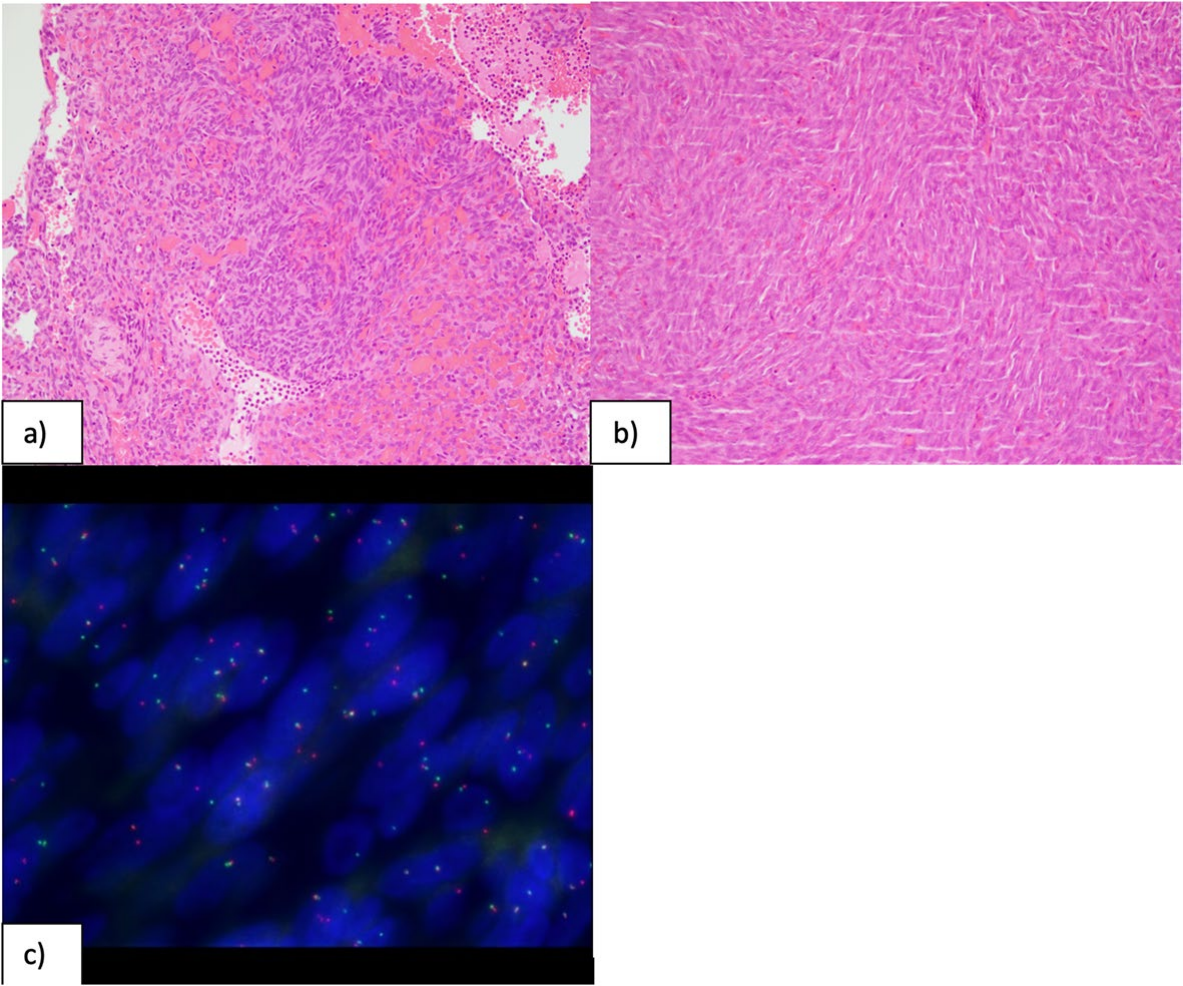
Images of the 2013 (**a**) and 2019 (**b**) tumour resections showing morphologically similar, mitotically active, spindle cell tumours (haematoxylin and eosin x200, microscope olympus bx53 with olympus dp27, camera and cellSens software to capture the images). FISH analysis of both tumours confirmed an SYT gene rearrangement (**c**). The normal signals consist of closely adjacent orange/red and green probes. The abnormal rearranged signal indicating SYT gene rearrangement is seen when there is a gap of at least twice the diameter of the fusion signal between the orange and green signal

Given the scan findings, the patient underwent resection of the recurrence with immediate post-operative MRI brain showing no residual tumour. Histological examination of the resection specimen showed a tumour consisting of short haphazard fascicles of monotonous basophilic spindle cells. Mitotic activity was readily identifiable with up to 24 mitoses per 10 high power fields. The tumour had a vaguely lobular architecture but no convincing meningothelial whorls or staghorn vessels were seen (Fig. 4). The tumour showed patchy positivity for EMA, bcl-2 and CD99. However, immunohistochemistry for STAT-6 and SSTR-2 were negative. On the basis of the morphology and immunohistochemical profile, fluorescent in-situ hybridisation (FISH) for *SS18 (SYT)* gene rearrangement was performed using an 18q11.2 *SS18 (SYT)* break-apart probe. This confirmed the presence of an *SS18* gene rearrangement consistent with synovial sarcoma (Fig. 4). On this basis, the FISH was repeated on the original 2013 tumour which also showed an *SS18* gene rearrangement consistent with the diagnosis of synovial sarcoma in the original tumour.

**Fig. 5:**
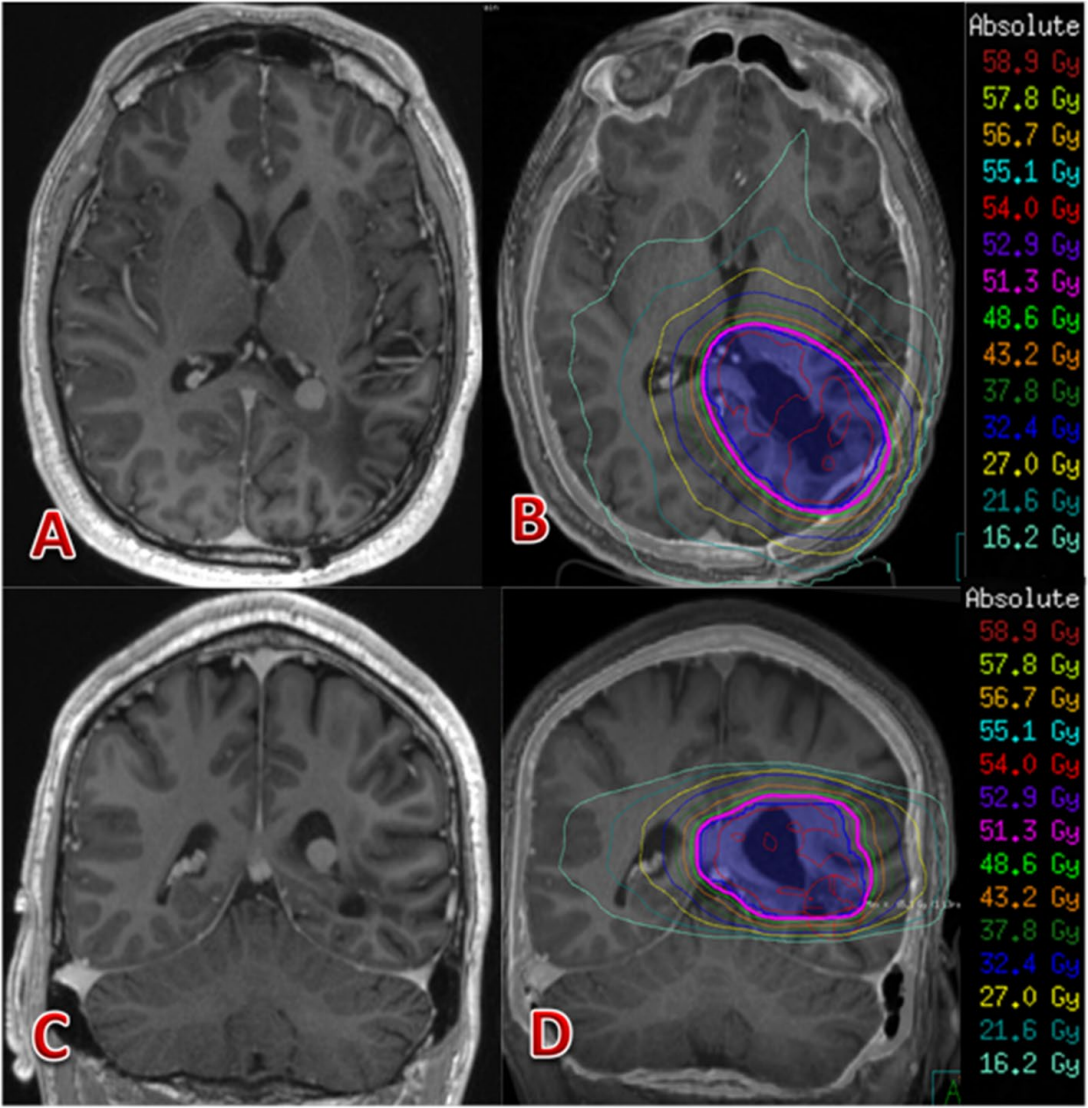
(**A**) and (**C**) showing planning MRI T1 post gadolinium contrast scans. **B** and (**D**) showing retreatment plan with bright pink line representing the PTV and surrounding isodose lines. PTV is the surgical bed with a margin allowing for microscopic disease extent and 0.3 mm departmental margin to allow for setup error

**Fig. 6:**
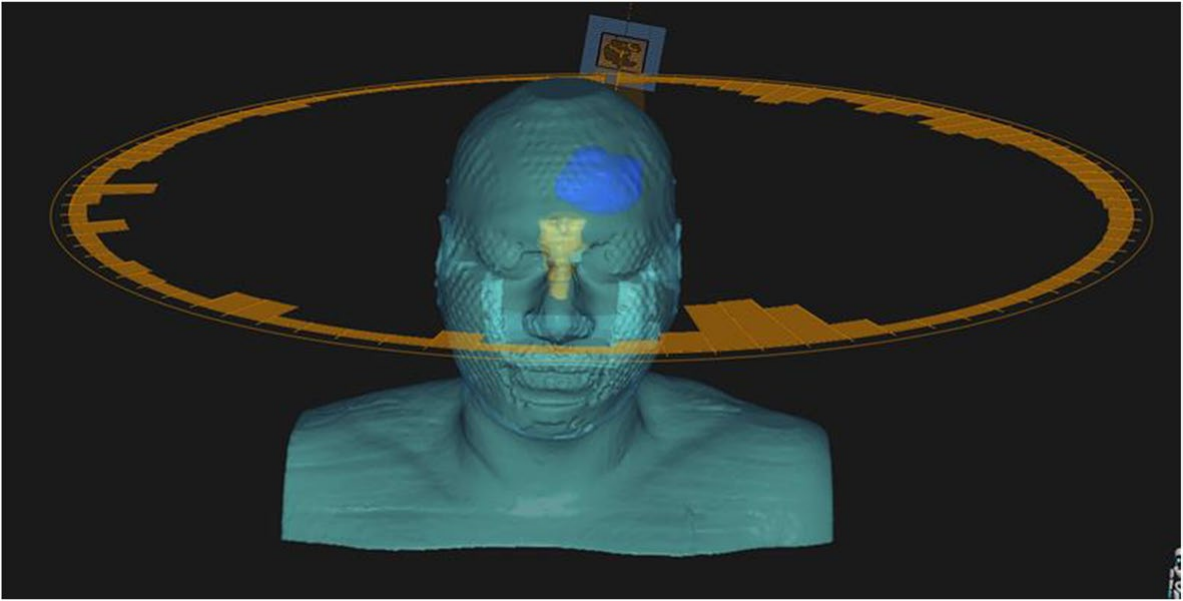
3D representation of the retreatment VMAT plan

Systemic staging showed no evidence of primary underlying malignancy or any sites of metastatic spread, and so the tumour was classified as a primary synovial sarcoma of the CNS. The patient was discussed at both the neuro-oncology and sarcoma tumour board multi-disciplinary meetings with recommendation for adjuvant radiation treatment. He proceeded with VMAT (Volumetric modulated arc therapy) retreatment to brain, 54 Gy in 30 fractions (see Figs. 5 and 6).

## Discussion and conclusion

Synovial sarcoma initially derived its name from a histologic resemblance to synovial cells [4]. There are two morphologic subtypes: monophasic and biphasic. Most synovial sarcomas are of the monophasic subtype and consist of relatively uniform spindle cells with hyperchromatic nuclei and sparse cytoplasm. Biphasic synovial sarcoma has an additional epithelial component of varying extent which may take the form of cells arranged in solid nests, cords, or glands [5]. The majority of synovial sarcomas are characterized by the chromosomal translocation t(X;18)(p11;q11). The breakpoint of this translocation fuses the *SS18* (previously called *SYT*) gene from chromosome 18 to one of three homologous genes, *SSX1*, *SSX2*, and *SSX4* on the X chromosome. The resulting fusion oncogene is thought to disrupt epigenetic control and mesenchymal differentiation through the SWI/SNF chromatin remodelling complex [6]. The exact cell of origin for synovial sarcoma remains to be determined. However, the two leading hypotheses are a mesenchymal stem cell found in the periosteum [7] or stem cells associated with neural crest cells [8]. Cytogenetic analysis, FISH, or RT-PCR can be used to detect the translocation or the protein product of the fusion gene, thus aiding in the diagnosis of synovial sarcoma [9].

Synovial sarcoma is associated with local recurrence and distant metastases. Metastases can occur in 50–70% of cases, with most metastases developing in the lung (80%) followed by bone (10%) and liver (5%) [10, 11]. Intracranial disease is rare but has been reported as metastasis from extracranial synovial sarcoma [12, 13]. Primary dural synovial sarcoma has also previously been reported [3]. We have not been able to identify a report of primary intraventricular sarcoma of the central nervous system in the literature using PubMed database. However intraventricular meningioma, which is thought to arise from meningothelial inclusions within the tela choroidea and mesenchymal stroma of the choroid plexus, is a well-recognised tumour entity [14]. It may be possible that a primary intraventricular synovial sarcoma (as in this case) arises from similar structures. In 2013 recurrent *NAB2-STAT6* fusions were identified as an almost pathognomonic finding in solitary fibrous tumour/haemangiopericytoma [15, 16]. Nuclear positivity for STAT6 by immunohistochemistry has since been established as a reliable surrogate of this fusion [17] and was invaluable in the current case in revising the diagnosis. The focal EMA positivity in this case also represented a potential pitfall which could result in the tumour being diagnosed as meningioma. However the negative SSTR2 immunohistochemistry in conjunction with the morphology prompted the consideration of synovial sarcoma. Surgical resection is the standard treatment for localized SS of the extremities, with consideration for use of neoadjuvant/adjuvant radiation and/or systemic anti-cancer therapy [18, 19]. Perioperative RT is associated with a statistically significant improvement in oncologic outcome among SS patient [20]. Prognosis for non-metastasized patients is often favourable for tumours < 5 cm resected with adequate margins [21, 22]. Given the paucity of data on primary intracranial synovial sarcoma, further research is needed to establish standard treatment.

This case report is significant for two reasons. Firstly, we believe this is the first reported case of primary intraventricular synovial sarcoma in the brain. Secondly, it highlights the need for extensive work up, including molecular testing where appropriate, of spindle cell tumours in the central nervous system. Synovial sarcoma represents a rare diagnostic pitfall that can mimic both hemangiopericytoma and meningioma – particularly when occurring at an unusual site. This is compounded by some overlap in the immunohistochemical profile of these tumours. In light of the ongoing significant advances in the molecular classification of all tumours, but CNS tumours in particular, patients with spindle cells tumours in the cranial cavity who present with recurrent disease should be reassessed with the full spectrum of immunohistochemical analysis and genetic testing necessary to ensure an up-to-date diagnosis.

## Data Availability

The analysed in this study is not publicly available due to patient confidentiality but will be available for sharing after local institutional ethics approval. Contact the corresponding author (Dr Anna McCool) if needed.

## Abbreviations

SS: Synovial sarcoma
MRI: Magnetic Resonance Imaging
WHO: World Health Organization
RT: Radiation therapy
VMAT: Volumetric modulated arc therapy
FISH: Fluorescent in-situ hybridisation
